# Segmental Distribution of Hepatocellular Carcinoma Correlates with Microvascular Invasion in Liver Explants Undergoing Transplantation

**DOI:** 10.1155/2019/8534372

**Published:** 2019-05-02

**Authors:** Yasir Al-Azzawi, Eva Rouanet, Ryan J. Hendrix, Lidia Spaho, Hesham Malik, Deepika Devuni, Gyongyi Szabo, Graham Barnard

**Affiliations:** ^1^Division of Gastroenterology, Department of Medicine, University of Massachusetts Medical School, Worcester, MA 01655, USA; ^2^Division of Surgical Oncology, Department of Surgery, University of Massachusetts Medical School, Worcester, MA 01655, USA; ^3^Department of Radiology, University of Massachusetts Medical School, Worcester, MA 01655, USA

## Abstract

**Introduction:**

Microvascular invasion (MVI) in hepatocellular carcinoma (HCC) patients is a poor prognostic factor after liver transplantation and/or resection. Any correlation between MVI and segmental location of HCC has yet to be studied. Our aim is to evaluate the segmental location of HCC and any correlation with the presence of MVI, portal vein thrombosis (PVT) in explanted livers, and the recurrence of HCC after transplantation. Another objective of the study is to assess the treatment history (ablation or transarterial chemoembolization (TACE)) and size of the tumor with respect to the risk of MVI.

**Methods:**

A single center, retrospective chart review, including 98 HCC patients, aged 18 years and older who had liver transplantation in our institute between 2012 and 2017. We reviewed the radiological images of the HCC tumors, the pathological findings of the explanted livers, and the follow-up imaging after transplantation.

**Results:**

98 patients with the diagnosis of HCC underwent liver transplantation between 2012 and 2017. The mean age of the cohort was 63 ± 8.2. Males represented 75% and Caucasian race represented 75% of the cohort. The most common etiology of cirrhosis was chronic hepatitis C virus infection followed by alcohol abuse and nonalcoholic steatohepatitis (NASH) with percentages of 50%, 23%, and 10%, respectively. Microvascular invasion was found in 16% of the patients while PVT and the recurrence of HCC were found in 17% and 6 % of the cohort, respectively. MVI was found in 10 single HCC and 6 multifocal HCC. Right lobe HCC had more MVI when compared to the left and multilobar HCC, with percentages of 11%, 2%, and 3%, respectively. Localization of HCC in segment 8 was associated with the highest percentage of MVI when compared to all other segments. The risk of MVI in segment 8 HCC was 3.5 times higher than the risk from the other segments (p=0.002) while no vascular invasion was found in segments 1, 3, and 5. The risk of vascular invasion in untreated HCC is 3 times the risk in treated HCC (P=0.03).

**Conclusion:**

Our data indicate that the risk of microvascular invasion is highest in tumors localized to segment 8. The size and number of HCC tumors were not associated with an increased risk of microvascular invasion.

## 1. Introduction

Hepatocellular carcinoma (HCC) is one of the most common malignancies worldwide and is the second leading cause of cancer-related deaths [1]. This results in over 600,00 deaths annually and a significant socioeconomic burden in the United States [2]. Furthermore, these numbers will continue to rise as HCC is currently considered as the fastest growing cause of cancer-related death in the United States with expectations of consistent growth over the next two decades [3, 4].

Even after potentially curative therapy with either hepatic resection or liver transplantation, tumor recurrence remains 70% at 5 years [5]. Microvascular invasion (MVI) has been identified as an independent predictor of tumor recurrence [6–8]. However, unlike macrovascular invasion which can be detected via radiological imaging, MVI is a histological diagnosis without universal diagnostic criteria [6]. Furthermore, the exact mechanism and implication of MVI for HCC is not well characterized.

Considering the difficulties of MVI identification prior to treatment and the strong effect on clinical outcomes, it is critical to characterize MVI in HCC for optimal management. Current approaches to characterize MVI have been targeted towards identifying prognostic factors and evaluation criteria, but none have attempted to associate the Couinaud segment predominance for HCC lesions with MVI [6, 9, 10]. Understanding the anatomical preferences and behavior within the liver could provide important observational and management information. In our study, we aim to identify whether HCC present in the various hepatic segments correlated with MVI in those particular hepatic segments.

## 2. Methods

### 2.1. Data Sources

We retrospectively reviewed the medical records of all patients over the age of 18 with pathology confirmed hepatocellular carcinoma who had a liver transplantation between January 1, 2012, and December 31, 2017, at the University of Massachusetts. By using the International Classification of Diseases (ICD) codes of hepatocellular carcinoma and liver transplantation, we were able to identify this patient's cohort. The study was approved by the Institutional Review Board of the University of Massachusetts Medical School and a HIPAA waiver of consent was granted given the retrospective and deidentified nature of the review. The chart of each patient was reviewed and the extracted data included the general demographic features of the cohort; the age, gender, and the race of the patients.

### 2.2. Patient Cohort

We performed a single center, retrospective chart review study of patients over the age of 18. Eighty-nine patients met the following inclusion criteria: a pathological finding of HCC in an explanted liver after liver transplantation at the University of Massachusetts Medical Center (UMMC). Postoperative recurrence of HCC was identified radiographically. The presence of PVT was assessed during the pretransplant period. Exclusion criteria were defined as loss to follow-up, liver biopsy, or liver transplant that was done outside the UMMS, incomplete records, and diagnoses other than hepatocellular carcinoma.

## 3. Results

Ninety-eight patients with a diagnosis of HCC underwent liver transplantation between 2012 and 2017. Baseline characteristics of patients are shown in Table 1. The mean age of the cohort was 63 ± 8.2. Males represented 75% of the cohort. Caucasian race represented 75% of the cohort. The most common etiology of cirrhosis was chronic hepatitis C virus infection followed by alcohol abuse and nonalcoholic steatohepatitis (NASH) with percentages of 50%, 23%, and 10%, respectively. From comparisons of patient characteristics according to the presence of MVI in the 98 explanted livers, MVI was found in 16 specimens (16.3%) with 10 being identified as single foci HCC and 6 as multifocal HCC. Portal vein thrombosis (PVT) was found in 17% of the cohort. Postoperative recurrence of HCC within the 5-year period of the study was found in 6% of the cohort (Table 1).

Further comparison of right and left lobe MVI showed a right-sided predominance when compared to left and multilobar HCC with percentages of 11%, 2%, and 3%, respectively (Table 2). We studied the association of the segment-specific location of the HCC and the presence of MVI which showed that localization of HCC in segment 8 was associated with the highest percentage of MVI when compared to all other segments. The risk of MVI in segment 8 was 3.5-times higher than the risk in other segments (p = 0.002) while no vascular invasion was found in segment 1, 3, or 5. To evaluate the association of the locoregional therapy with MVI, we compared the treated lesions with radiofrequency ablation and trans arterial chemo embolization to untreated HCC and found that the risk of MVI in untreated HCC is 3 times the risk in the treated HCC Odd ratio 3 and p=0.03.

## 4. Discussion

Liver cancers, which are predominately hepatocellular carcinoma, have high mortality rates and have been recently ranked as the second highest cancer-related death after lung cancer with a rising incidence in the last decade based on reports from the National Cancer Institute (NCI) [11, 12]. Treatment options for HCC depend mainly on two factors: the characteristic of the tumor in regard to size, number, and presence of vascular involvement and metastasis as well as the severity of the underlying liver disease and function as defined by the presence of portal hypertension and the general functional status of the patient. According to the American Cancer Society, the survival rates in patients with HCC using the TNM system depend on the stage of the disease. High 5-years survival rates (31%) were associated with localized cancer while low 5-year survival rates (3%) were associated with distant metastasis [13]. Expectedly, untreated HCC has a poor prognosis as defined by Giannini et al. who studied 600 patients with untreated HCC and found the 5-year survival to be 9.1% with a median survival duration in the advanced stages to be ranged between 6 and 7 months [14]. Surgical resection or liver transplantation remains cornerstones of therapy and despite the improvements in surgical techniques and perioperative care, long-term prognosis after surgical resection and transplantation remains unsatisfactory as the 5-year recurrence rate in resected HCC is estimated to be 70% after liver transplantation [15–17]. Given the ability to treat the underlying liver disease as well as the malignancy, liver transplantation is the favored procedure over resection.

Identification of prognostic factors after potentially curative resection or transplant has been evaluated to identify patients at greatest risk for recurrence of HCC. On multivariate analyses, the presence of microvascular metastasis has been identified as the strongest individual predictor of recurrence and survival in HCC patients [2]. In contrast to macroscopic vascular invasion, which is detectable with various imaging techniques and included as a diagnostic parameter in many HCC scoring systems, MVI is difficult to detect in the preoperative setting [18]. However, recent studies have supported its prognostic value in predicting risk of tumor recurrence and survival following potentially curative resection or transplant for HCC [8, 19–22]. Efforts to detect MVI status in the preoperative setting as a means to guide treatment have been attempted with diffusion weighted imaging (DWI), gadoxetic acid-enhanced magnetic resonance imaging (MRI), and ^18^F-fludeoxyglucose (FDG) positron emission tomography/computed tomography (PET/CT) [23, 24]. Reliable applications of these approaches have yet to be validated.

Multiple reviews have been conducted in an effort to identify the prevalence of MVI and to correlate MVI with tumor characteristics; however the results have been widely variable and conflicting. A systematic review by Zhang et al. found that the prevalence of MVI ranged widely from 15 to 57.1% [6]. Such a discrepancy can be explained by geographical variations and a lack of consensus regarding the definition of MVI [6]. They concluded that multinodular disease, HCC tumor size >4 centimeters (cm), and lymph node positivity were frequently associated with an increased risk of MVI [23]. Conversely, Jackhete et al. found that MVI was not associated with multilobar involvement in HCC, alpha fetoprotein (AFP) level, or the tumor differentiation [25] while Haung et al. found in a large cohort study that the MVI was associated with AFP>200, tumor encapsulation, tumor differentiation, and tumor size (>5cm) but no association was found with the gender, age, or etiology of the cirrhosis [26]. Our cohort supports Haung's findings as our study showed no association between MVI with the etiology of the cirrhosis, gender, or age. Our study did not show any association between the MVI and multilobar HCC. Upon comparing smaller tumor size (<2 cm) with larger tumor size (>=2 cm), our data showed that there is no association between the MVI and the size of the lesion, which contradicts Haung's result. This may be explained by a difference average tumor size as our cohort included only transplanted patients who, by Milan criteria, should have a tumor size less than 5 to be considered for liver transplantation.

As the prognostic value of preoperative identification of MVI has been clearly stated, we attempted to define a predictive relationship between the Couinaud segment localization of HCC and the subsequent risk for MVI. Of the 98 explanted livers evaluated for HCC, 16 (16%) were found to demonstrate MVI which is consistent with historical series. Stratifying by Couinaud segments, HCC tumors involving Couinaud segments 1, 3, 5, and 7 exclusively were not associated with any risk of MVI. Despite the small average size HCC in segment 8 (2.3cm), we found that it is a highly associated with the risk of MVI (OR 3.5, p=0.002). We found that the risk of MVI in segment 8 is three times the risk compared with other segments. Lesions located in segments 4, 5, and 8 are traditionally known as central HCC [27]. The unique anatomical characteristic of the central HCC's location, adjacent to the main hepatic artery vascular structure and having a dual blood supply from both left and right hepatic arteries, carries an increased risk of MVI [28]. Given that our data showed no correlation of MVI with tumor size and the overall smaller size of the lesions in segment 8, this supports an anatomical model for the increased risk of MVI in segment 8. Of note, a limitation of our study is that differences in tumor biology between segments were not compared and thus potentially more aggressive lesions in segment 8 may affect this model.

As mentioned above, one of the important predictors of recurrence is the presence of MVI and studies showed treating HCC with adjuvant therapy like TACE or RFA in addition to surgical resection extends patient survival [29]. Our data shows that locoregional therapy to HCC decreases the risk of MVI in the explanted livers and hypothetically decreases the risk of recurrence. The benefit of locoregional therapy, especially TACE, is debatable as Livoet et al. showed an increased survival rate in unresectable HCC when compared to the radical therapy while Oliveri et al. in his meta-analysis showed that there is no benefit of TACE or trans arterial embolization in unresectable HCC [30, 31]. Despite the average size of the HCC tumors in our cohort being relatively small, the locoregional therapy group showed a decrease in the risk of MVI in the explanted liver. The risk of MVI in untreated HCC is three time the risk in treated HCC, thus suggesting that even smaller sized lesions may still have MVI and may benefit from locoregional therapy either neoadjuvant or adjuvantly as it has been shown to decrease the risk of MVI.

## 5. Conclusion

Our data indicate that the risk of microvascular invasion is highest in HCC tumors located in Couinaud segment 8. The size and the number of HCC tumors were not associated with an increased risk of microvascular invasion in our series of patients. Locoregional therapy whether alone, adjuvant, or a bridge to transplantation can decrease the risk of MVI. We recommend a larger and prospective study to investigate the effect of the locoregional therapy in decreasing the risk of MVI.

## Figures and Tables

**Table 1:** Baseline characteristics of the study cohort.

Characteristics	Number (%)
Age	63 +/- 8.2
Gender	
Male	74(75%)
Female	24(25%)
Race	
Caucasian	75 (75%)
African-American	4(4%)
MELD	
HCV	50 (50%
HBV	9(9%)
Alcohol	23(23%)
NASH	10 (10%)
CTP A	24(24%)
CTP B	35 (35%)
CTP C	39 (39%)
Multifocal	46 (46%)
Single	52 (52%)
Segment 1	1 (1%)
Segment 2	4 (4%)
Segment 3	0 (0%)
Segment 4	5 (5%)
Segment 5	6 (4%)
Segment 6	6 (6%)
Segment 7	7 (7%)
Segment 8	18 (18%)
Undetermined	5 (5%)
Vascular Invasion	16 (16%)
Recurrence	6 (6%)
PVT	17 (17%)

**Table 2:** Odds ratios of microvascular invasion.

Characteristics	Microvascular Invasion	Odds Ratio	p-value
Single	10	1.5	0.3
Multifocal	6	0.6	0.3
Right Side	11	1.2	0.7
Left Side	2	0.6	0.3
Multiple Lobes	3	1.2	0.7
Single Lobe			
Segment 1	0	0	0
Segment 2	1	1.7	1
Segment 3	0	0	0
Segment 4	1	1.3	0.7
Segment 5	0	0	0
Segment 6	2	2.0	0.19
Segment 7	0	0	0
Segment 8	6	3.5	0.002
Disease Etiology			
HCV	8	1	1
EtOH	3	0.7	0.5
NASH	3	2.4	0.2
Tumor Size			
Size > 2cm	10	0.4	0.6
Size < 2cm	6	1.2	0.6
Untreated	10	3.0	0.03
Treated	6	0.3	0.03

## Data Availability

The data for this research was provided by the University of Massachusetts Medical Center.

## Additional Points

*Limitations. *Our study has the limitations inherent to a retrospective review of data, and a selection bias cannot be excluded because we included only transplanted patients. While all data were recorded prospectively and in real-time, there was no treatment or intervention arm, thus direct cause and effect conclusions cannot be drawn. Comparison of our data to historical series is limited given the heterogeneity of study populations and lack of consistency in anatomic definitions of MVI. Further randomized controlled trials are needed in the future to more thoroughly address these questions.
